# Derotational Corrective Osteotomy for Pediatric Forearm Malunion: A Case Report

**DOI:** 10.7759/cureus.72574

**Published:** 2024-10-28

**Authors:** Paula Vieira, João Lixa, Carlos Lobão, João Carvalho, Joana Pereira

**Affiliations:** 1 Orthopedics and Traumatology, São João University Hospital Center, Porto, PRT; 2 Orthopedics and Traumatology, Tâmega e Sousa Hospital Centre, Porto, PRT

**Keywords:** 3d ct scan, 3d printed template, derotational osteotomy, forearm fracture malunion, forearm rotational deformity, pediatric forearm fractures

## Abstract

In this case report, we describe an eight-year-old boy with both-bone forearm diaphyseal fracture that was treated conservatively after closed reduction with manipulation. Nine months after the injury, he returned to consultation, presenting a rotational deformity of the forearm and 20° of pronosupination limitation. He was submitted to corrective osteotomies, using three-dimensional (3D) planning and templating, using a double approach, and fixation with a four screw holes plate in each osteotomy. At one year of follow-up, there are signs of bone consolidation and complete recovery of range of motion.

## Introduction

Forearm fractures are among the most common pediatric injuries, accounting for about 5-10% of all pediatric fractures [1,2], typically resulting from a fall with an outstretched hand [1,3]. These fractures are usually managed non-operatively with closed reduction and cast immobilization with good results, especially in children younger than 10 years old. Despite radiographic malunion being common, reaching up to 40%, potential remodeling by bone growth makes symptoms occur in less than 0.5% of the cases [3,4]. In case of symptomatic malunion, corrective osteotomy is indicated, with several techniques described in the literature, like minimally invasive drill osteoclasis or open osteotomy (conventional or three-dimensional (3D) planned) [3]. Minimally invasive techniques allow for angular deformity correction, avoiding internal fixation and a second procedure for hardware removal. However, the results for rotational and multiplanar deformities are unclear. In these cases, open osteotomy techniques with plating are recommended [3].

Deformity analysis and correction are complex and require preoperative planning. 3D analysis and 3D-printed templates have seen increased interest in recent years, as they permit a better understanding of the deformity and surgical planning, shortening operative time [5]. However, it remains unclear whether the outcome justifies the additional cost and radiation exposure associated with 3D-planned patient-specific techniques [4-7].

We present a case of an eight-year-old boy with symptomatic malunion submitted to corrective osteotomy for both-bone forearm fracture using a 3D-printed template.

## Case presentation

**Figure 1:**
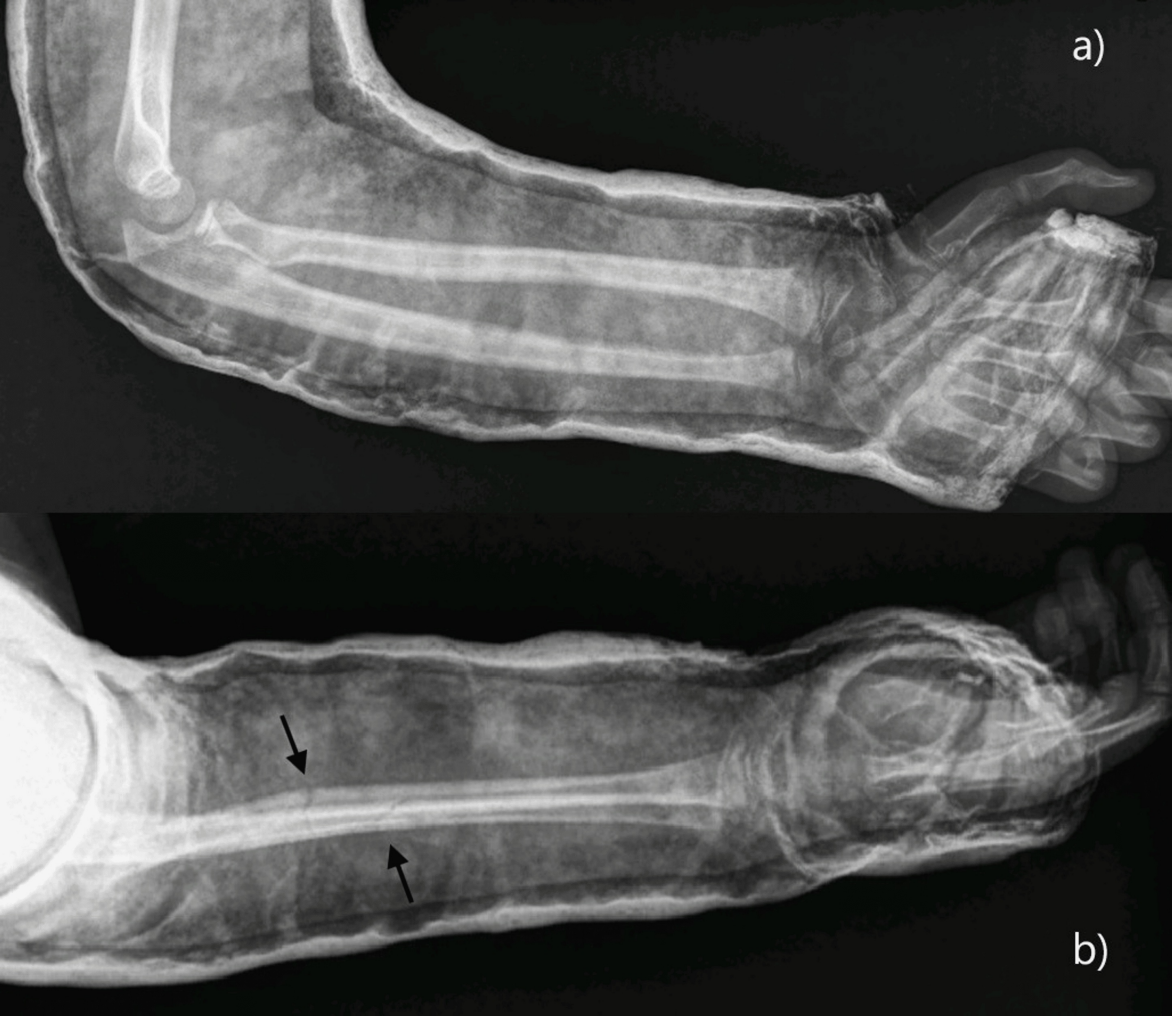
Forearm radiograph post-closed reduction a) Anteroposterior view; b) side view.

An eight-year-old Caucasian boy with no significant past medical or surgical history presented at the emergency department with right forearm pain after a fall. The radiograph showed a complete both-bone forearm fracture in the middle diaphyseal third, with good alignment post-closed reduction (Figure 1), which was treated conservatively with a long arm cast for four weeks and then a forearm cast for another four weeks.

At the end of the immobilization, he presented good callus formation on the radiograph, with a radial angulation of 14° on the anteroposterior view, 12° on the side view, a cubitus angulation of 6° on the anteroposterior view, and 2° on the side view. From a clinical point of view, he presented painless and with progressive improvements in his range of motion, having lost follow-up after referral to physical therapy.

**Figure 2:**
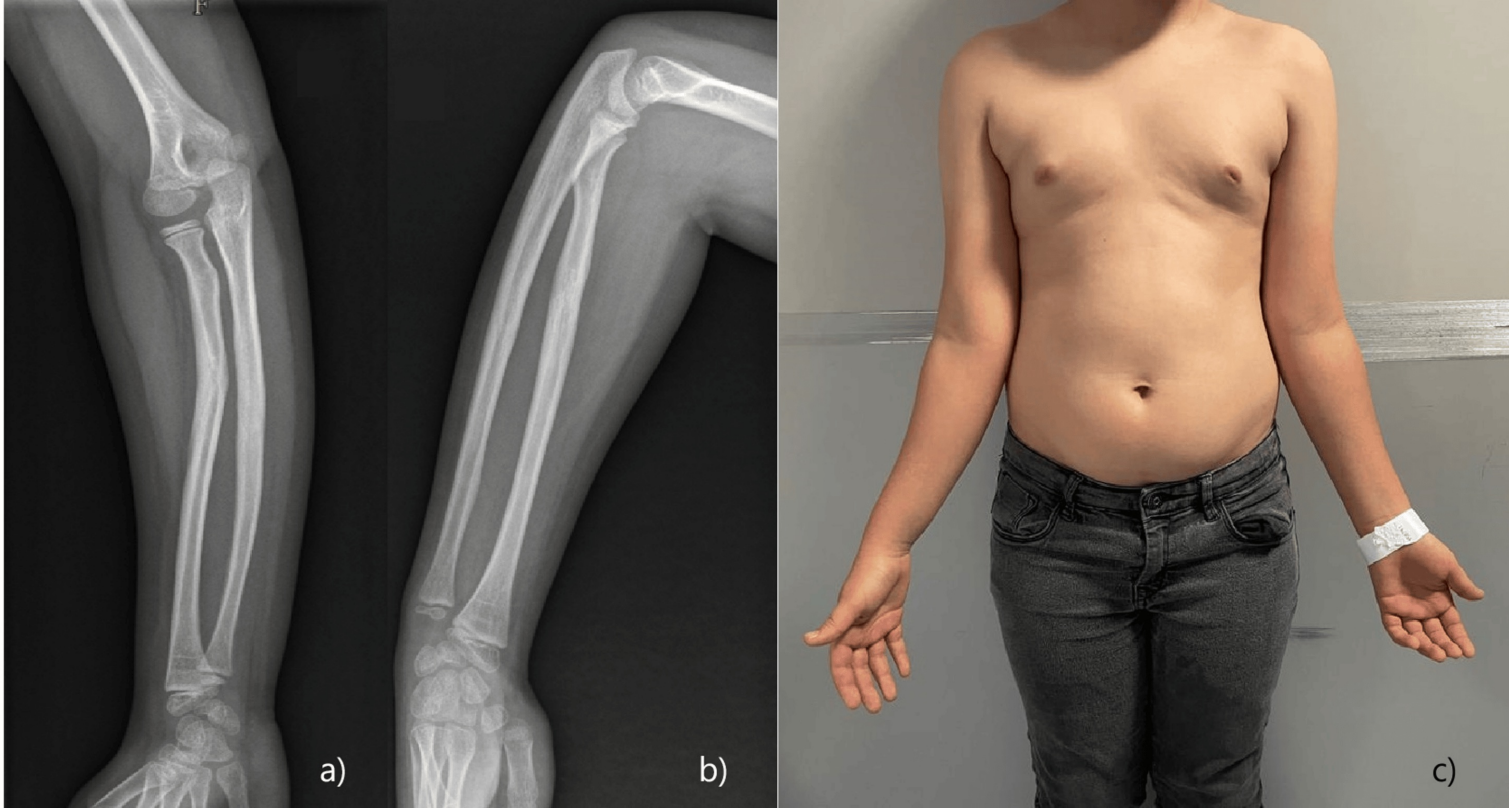
Re-evaluation at nine months after the injury a) Anteroposterior view of forearm radiograph; b) side view of forearm radiograph; c) physical examination with right arm rotational deformity and supination limitation.

Nine months after the injury, he returned for an orthopedic consultation. The radiograph showed signs of consolidation of both fractures, with an angular and rotational deformity (Figure 2). He presented signs of distal radioulnar joint (DRUJ) instability, with associated crepitus and a reduction of 20° of prono-supination (Figure 2).

Due to the limitation of the range of motion and deformity, surgical treatment was proposed.

Preoperative planning and surgical technique

Magnetic resonance imaging (MRI) and computed tomography (CT) studies were performed to evaluate elbow and wrist joints associated lesions and 3D planning of the deformity. DRUJ and elbow joint presented without any significant alterations. Full-length AP and lateral radiographs of contralateral forearm were also obtained to help anticipate the degree of correction.

Being a multiplanar injury with rotational deformity, percutaneous techniques were ruled out. An open osteotomy technique was proposed. A 3D-printed plastic template was used to determine the amount of derotational correction needed, as well as to determine the level and orientation of the ulna and radius osteotomies.

The patient was operated on in the supine position; the ulnar side was approached first by a direct approach between muscle flexor carpi ulnaris and muscle extensor carpi ulnaris. A transverse osteotomy was done, the anatomic relationships of the coronoid process and ulnar styloid were restored, and provisional K-wire fixation was performed. The radius was then approached by a volar (Henry) approach at the level of the fracture site, recurring to a closing wedge osteotomy at the apex of the angulation and derotation under fluoroscopy control, restoring the radial tuberosity and radial styloid relationship. A full range of pronosupination and flexion/extension was tested before definitive fixation with a four screw holes plate in each osteotomy.

**Figure 3:**
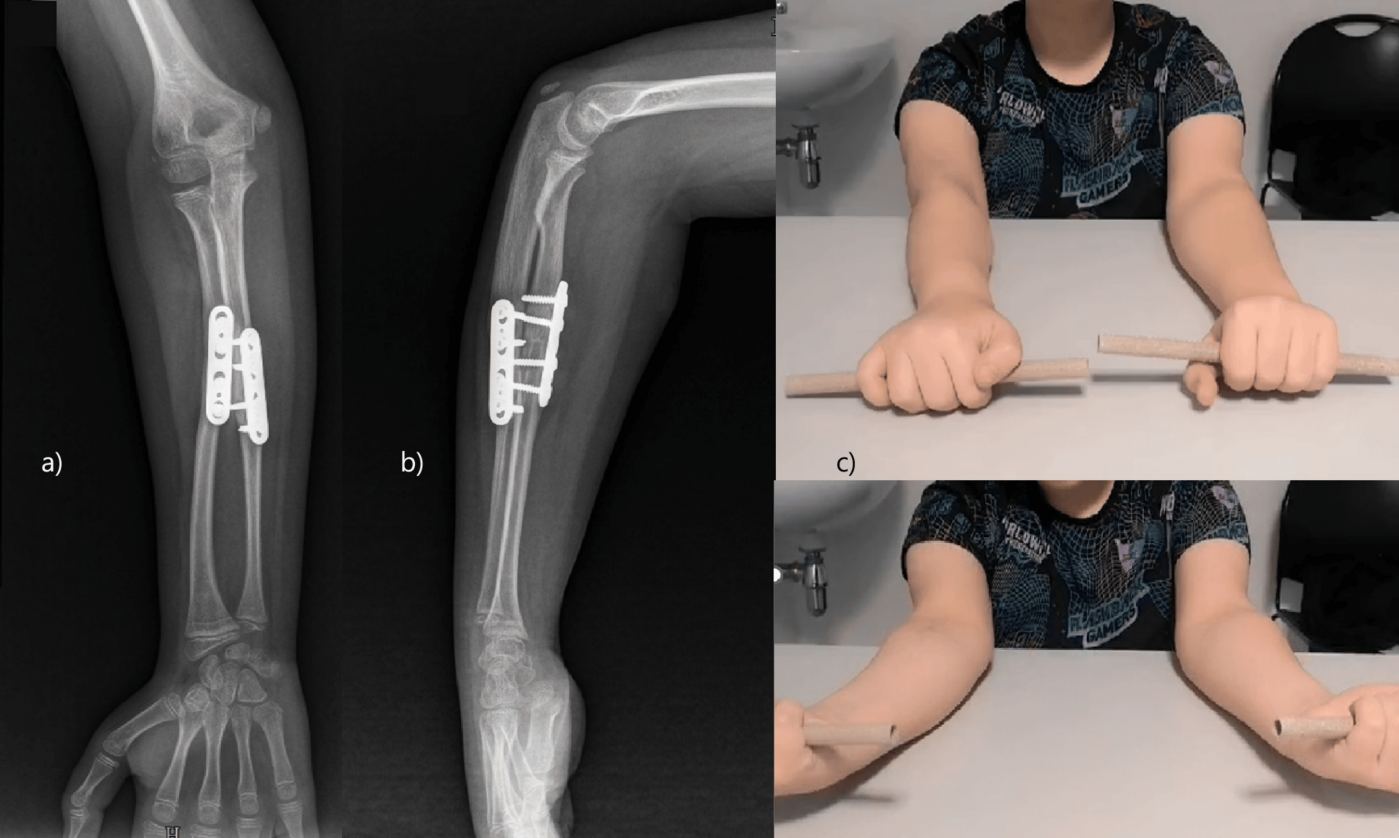
Re-evaluation at one year after surgical procedure a) Anteroposterior view of forearm radiograph; b) side view of forearm radiograph; c) physical examination with pronosupination symmetry.

A long arm cast immobilization was used for two weeks postoperatively, followed by a range of motion rehabilitation. At one year of follow-up, the patient presented as asymptomatic, with a complete range of motion (Figure 3), and with no signs of surgical complications. The osteotomies show signs of consolidation (Figure 3), the rotational deformity is corrected, and he has no limitation in sporting activities.

## Discussion

The remodeling potential of fractures in children is a unique phenomenon that allows many pediatric fractures to be treated non-operatively. Even high angulated diaphyseal fracture, which may lead to a persistent radiographic deformity, can result in a realignment to the limb axis [8]. 

Bone remodeling capacity is inversely proportionate to the child’s age [8]. In relation to the forearm, proximal fractures like the one seen in this case have less remodeling capacity since radial and ulnar growth occurs 75-80% in the distal physis [9]. Several criteria and angulations have been proposed for guidance in the conservative treatment of pediatric forearm fractures [2,9]. Noonan and Price recommended that in children <9 years, 15° of angulation and 45° of malrotation is acceptable, but in children >9 years with proximal forearm fractures, only 10° of angulation and 30° of malrotation can be accepted [9].

The biomechanics of forearm motion depend on a complex interplay between the radial bow, the ulna, the interosseous membrane, and the DRUJ. In the diaphysis, the interosseous distance is greatest, which may justify that a shaft fracture can cause more mechanic alterations [10]. Altered biomechanics can lead to long term osteoarthritis [4] besides cosmetic deformities and painful motion.

Literature states that 50° of supination and pronation (100° of range of motion for the forearm) are sufficient for activities of daily living and that in pediatric patients, the compensating mechanisms are even higher [11]. Thus, symptomatic malunion of both-bone forearm fractures in children under the age of 10 years old is rare [3,4]. However, there is a lack of high-quality studies regarding the treatment of symptomatic malunion in these patients.

Proposed indications for corrective osteotomy are functional impairment, pain at the DRUJ with motion, or unacceptable forearm deformity [12]. The patient presented had a diaphyseal forearm fracture, which, although initially met the acceptable criteria for conservative treatment, later progressed to unacceptable rotational deformity with DRUJ pain and pronosupination impairment. Although his young age provided some potential for bone remodeling, the proximal location of the fracture was a risk factor for malunion.

Various corrective osteotomies have been described as effective in treating malunion [2,3], with good functional results [13]. Techniques ranging from percutaneous osteoclasis to open conventional osteotomy to the utilization of patient-specific instrumentation (PSI) recurring to 3D-planning and printing [1,3-7,12,13]. For rotational and multiplanar deformities, open osteotomy techniques are recommended. Although requiring greater surgical exposure and internal fixation (which often requires surgical removal), open osteotomy and plating allow for complete recovery of DRUJ stability and forearm rotation [3]. PSI and 3D planning can help to better understand the deformity, reducing surgical time (32 minutes shorter on average), complexity, and incision size, permitting a more anatomical correction [3-7]. The additional radiation necessary for forearm CT acquisition and high materials costs (up to $4300 per case) are the main disadvantages of this technique [1,3]. In our case, we planned open osteotomies, using 3D-planning in a 3D-printed forearm CT reconstruction of the affected side, as PSI was not available in our Institution. The osteotomies were performed in the template, and a correction was simulated.

Regarding osteotomy sequence, the ulnar side is usually approached first as limited supination may hinder a volar approach to the radius [3,12,13], such as the sequence that was used in our case.

In a recent meta-analysis of functional outcomes after corrective osteotomy (conventional or 3D assisted) for pediatric forearm fractures malunion, Roth et al. [14] reported a mean gain in forearm rotation of 77° after surgery, with greater correction in children younger than 13 years old (87° vs. 68°). They also found that correction was superior in patients operated on within one year after trauma (93° vs. 61°). These results are similar to the reported by van Geenen et al. [12] in their series of 21 malunions. In fact, several authors suggest optimal timing for osteotomy within one year of the injury, before the occurrence of joint changes and soft-tissue contractures [12-14]. Our patient was submitted to deformity correction before 10 years old and before one year of the fracture, probably contributing to a better outcome and a complete restoration of range of motion.

## Conclusions

Although rare, forearm malunions in the pediatric age may lead to important complications, like deformity and motion impairment, as seen in this case. These injuries should have a close follow-up even after the end of immobilization, as complications may have better outcomes if diagnosed and treated within one year of injury. Corrective osteotomies show promising results, which can be facilitated by 3D technology. Our patient was submitted for deformity correction within one year after the fracture using 3D planning and 3D printing templates. At one year of follow-up, he was asymptomatic and had a complete range of motion.
